# Network Mechanisms Generating Abnormal and Normal Hippocampal High-Frequency Oscillations: A Computational Analysis^1^,^2^,^3^

**DOI:** 10.1523/ENEURO.0024-15.2015

**Published:** 2015-06-24

**Authors:** Christian G. Fink, Stephen Gliske, Nicholas Catoni, William C. Stacey

**Affiliations:** 1 Department of Physics & Astronomy and Neuroscience Program, Ohio Wesleyan University, Delaware, Ohio 43015; 2 Department of Neurology, University of Michigan, Ann Arbor, Michigan 48109; 3 Department of Neuroscience, Brown University, Providence, Rhode Island 02912; 4 Department of Biomedical Engineering, University of Michigan, Ann Arbor, Michigan 48109

**Keywords:** fast ripple, high-frequency oscillation (HFO), hippocampus, rhythmogenesis, ripple, synchronization

## Abstract

High-frequency oscillations (HFOs) are an intriguing potential biomarker for epilepsy, typically categorized according to peak frequency as either ripples (100–250 Hz) or fast ripples (>250 Hz). In the hippocampus, fast ripples were originally thought to be more specific to epileptic tissue, but it is still very difficult to distinguish which HFOs are caused by normal versus pathological brain activity. In this study, we use a computational model of hippocampus to investigate possible network mechanisms underpinning normal ripples, pathological ripples, and fast ripples. Our results unify several prior findings regarding HFO mechanisms, and also make several new predictions regarding abnormal HFOs. We show that HFOs are generic, emergent phenomena whose characteristics reflect a wide range of connectivity and network input. Although produced by different mechanisms, both normal and abnormal HFOs generate similar ripple frequencies, underscoring that peak frequency is unable to distinguish the two. Abnormal ripples are generic phenomena that arise when input to pyramidal cells overcomes network inhibition, resulting in high-frequency, uncoordinated firing. In addition, fast ripples transiently and sporadically arise from the precise conditions that produce abnormal ripples. Lastly, we show that such abnormal conditions do not require any specific network structure to produce coherent HFOs, as even completely asynchronous activity is capable of producing abnormal ripples and fast ripples in this manner. These results provide a generic, network-based explanation for the link between pathological ripples and fast ripples, and a unifying description for the entire spectrum from normal ripples to pathological fast ripples.

## Significance Statement

Approximately 0.25% of people throughout the world suffer from uncontrolled epilepsy, largely due to our incomplete understanding of how seizures are generated. This motivates the search for new epilepsy biomarkers, one of the most promising of which are high-frequency oscillations (HFOs): focal, brief field potential signals of 80 Hz or more. Not all HFOs are pathological, however, and despite 20 years of research, it is still unclear how to distinguish normal from pathological HFOs. We use a computational model to investigate the network properties capable of generating two types of HFOs, ripples and fast ripples. Our model indicates that a range of physiological conditions are capable of producing the full spectrum of HFOs, from normal ripples to “epileptic” fast ripples.

## Introduction

High-frequency oscillations (HFOs) have attracted much attention over the past several years as a potential biomarker of epileptic tissue. HFOs are brief oscillations of the local field potential (usually <100 ms) over 80 Hz that stand out from background. They were originally discovered in the CA1 region of normal hippocampus (Buzsáki, 1986; Buzsaki et al., 1992) and called “ripples” (<250 Hz). Bragin et al. (1999) subsequently found that HFOs were increased in epileptic hippocampus in humans. They also identified a new class of faster oscillations (>250 Hz), termed “fast ripples.” Since that time, much effort has focused on characterizing the role of HFOs in epilepsy (Jacobs et al., 2012).

Although these studies suggest the potential of HFOs as a novel epilepsy biomarker, subsequent human studies have demonstrated the difficulty in determining whether a given HFO stems from normal or epileptic processes (Engel et al., 2009; Kerber et al., 2014). Most clinical studies have recorded HFOs using macroelectrodes (Jirsch et al., 2006; Urrestarazu et al., 2007), and some studies using microelectrodes have found that some HFOs are detected more accurately using higher resolution (Bragin et al., 2002b; Le Van Quyen et al., 2008; Worrell et al., 2008). Both ripples and fast ripples are increased in epileptic tissue (Jirsch et al., 2006; Urrestarazu et al., 2007), though the ratio between them is altered in epilepsy (Staba et al., 2007). Fast ripples are seen in normal neocortex (Jones et al., 2000; Coppola et al., 2005) and have recently been recorded in hippocampal tissue that does not participate in seizures (Kucewicz et al., 2014), thus illustrating the need to better understand the mechanisms underpinning different varieties of HFOs (Jefferys et al., 2012).

Initial studies indicated that normal and epileptic HFOs are produced by different mechanisms. Ripples are formed in normal tissue by IPSPs when interneurons fire in-phase with the oscillation and pyramidal cells fire very sparsely (Ylinen et al., 1995; Csicsvari et al., 1999). Subsequent computational studies have further bolstered this finding (Taxidis et al., 2012; Brunel and Wang, 2003). In contrast, large numbers of pyramidal cells become active during pathological HFOs (Bragin et al., 2011). It is currently unclear exactly how networks produce fast ripples. Proposed mechanisms include networks of axo-axonal gap junctions (Traub et al., 2005; Roopun et al., 2010), recurrent synapses between pyramidal cells (Dzhala and Staley, 2004), asynchronous input from CA3 to CA1 regions in the hippocampus (Demont-Guignard et al., 2012), and reduced spike-time precision resulting in the emergence of two out-of-phase clusters (Foffani et al., 2007; Ibarz et al., 2010). Although each of these hypotheses has merit, they have been difficult to reconcile and test experimentally due to limitations in available recording technology. In addition, each of the above theories is subject to important constraints upon the network—in each case, the fast ripples arise only under specific conditions.

In this paper, we develop a computational model of hippocampus with the goal of determining which network phenomena are necessary and/or sufficient to produce normal ripples, pathological ripples, and fast ripples, as well as to explore mechanistic links between these rhythms. We use a physiologically realistic model of hippocampus (the “biophysical model”) in which we vary two generic network properties: the number of inhibitory connections and the intensity of excitatory input to all cells. This model allowed exploration of generic network effects on HFOs. However, given the remarkable capacity for distinct mechanisms to generate similar HFOs, we also explored how HFOs may arise generally, independent of any specific network structure. In essence, an HFO is produced by the summation of IPSPs or action potentials (APs) recorded at the electrode. Therefore, we also develop a constructed local field potential (“constructed LFP”) model that explicitly controls when IPSP and AP waveforms occur, without any specific network structure. This constructed LFP model enables exploration of generic network properties necessary to generate HFOs, such as synchronous versus asynchronous firing.

We show that HFOs are an emergent phenomenon produced over a broad range of connectivity structures and levels of synaptic input. Although similar results have been demonstrated in models of normal HFOs, our model produces the full spectrum from gamma frequencies to fast ripples, and uncovers several novel characteristics of epileptic HFOs. First, the model predicts that HFOs in the ripple range can be produced by either epileptic (i.e., APs; Bragin et al., 2011) or normal (i.e., IPSPs; Ylinen et al., 1995) mechanisms, and that peak frequency is unable to distinguish between the two. Second, we show that fast ripples are generic phenomena that are generated by APs and arise when synaptic input overcomes network inhibition enough to allow out of phase firing. Third, ripples produced by APs are prone to transient shifts into fast ripples, which may explain why fast ripples are often inconsistent in experimental recordings. Finally, we show that HFOs are a generic property of active neural populations and can be generated without any specific network structure, even with completely asynchronous activity.

## Models

### Biophysical Model

Our biophysical model of hippocampus was simulated using NEURON 7.3 (Hines and Carnevale, 1997) and is based upon two previously published models of hippocampal oscillations. The first described the interplay between gamma and theta oscillations in normal hippocampus due to feedback inhibition from basket and oriens lacunosum moleculare (OLM) cells (Tort et al., 2007). The second adapted the same network structure to demonstrate how ripples (<250 Hz) arise when epileptic pathologies are present in the network, based upon the relationship between inhibitory interneurons, network connectivity, and synaptic drive to the pyramidal cells (Stacey et al., 2009). This latter study did not include the OLM cells, because they only affected the much slower theta (<10 Hz) frequencies in the first model.

Both of those models used blocks of 80 pyramidal cells with 20 basket cells, and the output generated from the membrane voltages of each cell. In the current model, all cellular and synaptic parameters are identical to the previous work (Tort et al., 2007; Stacey et al., 2009). Each pyramidal cell has five compartments (basal dendrite, soma, and a three-compartment apical dendrite) and the basket cells have a three-compartment soma. We made two alterations to the model. All cells are given three-dimensional coordinates as a two-layer planar disk placed 50 μm from a recording electrode (see below), and the number of pyramidal cells is increased to 3080.

**Figure 1:**
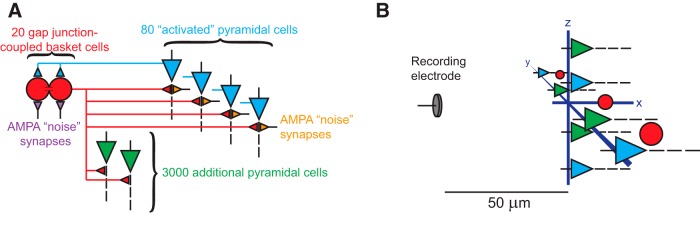
Schematic of the computational model of hippocampus. ***A***, The model consisted of 3080 pyramidal cells and 20 basket cells. Eighty of the pyramidal cells received noisy synaptic input that excited the cells and could produce action potentials. Each basket cell was coupled with gap junctions to the nearest neighboring basket cells, and each sent GABAergic connections to all pyramidal cells. Basket cells received feedback AMPAergic connections from activated pyramidal cells, and they also received noisy synaptic input. All noisy input was independent from cell to cell throughout the network. ***B***, All cells were distributed uniformly along two perpendicular axes in a plane 50 μm from the simulated recording electrode. The furthest cells were located 215 μm from the recording electrode. The voltage recorded by the electrode was generated by summing the voltage produced by the transmembrane current from every compartment of every neuron and interneuron.

In this simulation, 80 of the pyramidal cells are actively being driven by excitatory afferent synapses, representing a small cluster of “activated” cells within a larger network; the remaining 3000 cells receive no excitatory input. As shown in Figure 1, all pyramidal cells receive GABAergic synapses from all 20 basket cells. Each of the activated pyramidal cells has efferent AMPAergic synapses with on average two or three basket cells. The other 3000 cells never fire APs because their only input is inhibitory, so efferent synapses are unnecessary; their role in the simulation is to demonstrate the effect of the divergent IPSPs from the basket cells. Basket cells send efferent GABAergic synapses to all 3080 pyramidal cells (*τ*_rise_ = 1.5 ms, *τ*_decay_ = 8.0 ms, *g*_max_ = 5.5 nS, *E*_rev_ = −80 mV), receive AMPAergic synapses from 10 activated pyramidal cells (*τ*_rise_ = 0.2 ms, *τ*_decay_ = 1.0 ms, *g*_max_ = 0.5 mS/cm^2^, *E*_rev_ = 0 mV; Tort et al., 2007; Stacey et al., 2009), and are coupled to each other with somatic gap junctions, as seen experimentally wherein they form a synchronous syncytium (Amitai et al., 2002). Thus, the basic connectivity of this model consists only of the inhibitory feedback between pyramidal and basket cells. This reduced structure assures the model is restricted to phenomena present within this generic connectivity.

The only driving input to the model simulates the primary excitation present *in vivo*: afferent synaptic activity. From the point of view of each cell, synaptic inputs arriving from different brain regions can be modeled as random events, or “synaptic noise.” Synaptic noise was previously shown to be capable of producing HFOs (Stacey et al., 2009), and was recently shown to provoke seizures *in vitro* (Jirsa et al., 2014). Thus, the afferent activity on both basket and pyramidal cells was modulated by varying the intensity of AMPA “noise” synapses. Only 80 of the pyramidal cells were activated by this noise. For each noise synapse, the time between subsequent synaptic events followed an exponential distribution, so that the arrival of synaptic noise events was a Poisson process, independent from cell to cell. The mean of this distribution determined the overall noise intensity, with smaller mean interevent interval implying greater intensity. For low intensities it has already been shown that the model generates gamma oscillations (Tort et al., 2007), typical of the PING phenomenon (Traub et al., 1997). In this work, we describe how the peak frequency of the network LFP output increases accordingly as synaptic drive increases, so that the model produces the full spectrum of fast oscillations: gamma, fast gamma, ripples, and fast ripples.

This model enabled the simulation of sharp wave ripples by increasing the intensity of synaptic noise received by either pyramidal cells or basket cells, in a manner similar to the mean-field model of Demont-Guignard et al. (2012). Simulated sharp waves lasted for 35 ms in our model, with onset and offset following a Gaussian distribution (*σ* = 7 ms) across the neuronal population (to reproduce the physiological appearance of sharp waves and avoid nonphysiological, hypersynchronous onset).

The LFP recorded from neural activity was simulated by determining the voltage seen by an ideal microelectrode due to the transmembrane current from every compartment of every cell. This was done by recording the transmembrane current in all *N* compartments (Malmivuo and Plonsey, 1995) and calculating the following:

(1) $$V({\vec{r}}_{e},t)=\frac{\rho }{4\pi }\sum_{j=1}^{N}\frac{I_{j}(t)}{\left|{\vec{r}}_{e}-{\vec{r}}_{j}\right|},$$

where $V({\vec{r}}_{e},t)$ is the net electric potential at the recording electrode at time *t*, *ρ* is the extracellular resistivity, *I_j_* is the transmembrane current in compartment *j*, and $\left|{\vec{r}}_{e}-{\vec{r}}_{j}\right|$ is the distance between compartment *j* and the recording electrode (these distances ranged from 50 to 215 μm). The quantity *ρ* was set to 351 Ω · cm (Latikka et al., 2001), and all neurons were located in a plane whose closest point was 50 μm from the simulated recording electrode (see Fig. 1*B* for a schematic of the spatial arrangement of the network and recording electrode). NEURON code for the model is available in ModelDB (Hines et al., 2004), accession number 182134.

### Constructed LFP Model

One major goal of this work is to determine the generic mechanisms that produce epileptic and normal HFOs. We sought to answer, independent of any network structure, what type of activity is necessary and sufficient to produce each type of HFO. As it is impossible to simulate all potential network configurations, we developed a more basic method of producing neural signals. We explicitly defined the onset times for a large number of either IPSP or AP waveforms (results shown in [Fig F8][Fig F9][Fig F10]). This model did not include any neuronal structure; it was simply a mathematical reconstruction of a number of IPSP or AP waveforms, using the same waveforms generated by the biophysical model. The goal of this model was to show, under completely controlled conditions, how the LFP would appear if it were generated purely by either type of waveform. The model allowed an explicit demonstration of the differences between these two cases, and also enabled exploration of the relationship between variability in cell firing and network output. To generate this output, we recorded from 200 μm away the LFP voltage produced by an AP in a single pyramidal cell in our biophysical model, as well as that produced by a basket cell IPSP onto a pyramidal cell. These two waveforms, which we denote *h*_AP_(*t*) and *h*_PSP_(*t*), were used as templates for the output of each AP or IPSP. We then simulated a population of cells producing these waveforms at specific times using the process described below. The AP waveform had an amplitude of 0.383 μV and a full-width at half-max duration of 0.65 ms, whereas the PSP waveform had corresponding values of 0.0237 μV and 15.3 ms. These parameters are consistent with those reported in previous studies (Gold et al., 2006; Bazelot et al., 2010).

Two different statistical procedures (described shortly) were used to generate a sequence of event times (modeled as Dirac delta functions) for each of *N* neurons, with each event representing the trigger time of either an AP or IPSP:

(2) $$s_{i}(t)=\sum_{j=1}^{n_{i}}\delta (t-t_{i}^{j}),$$

where *s_i_*(*t*) is the event sequence of the *i*th neuron, *n_i_* is the total number of events of the *i*th neuron, and $t_{i}^{j}$ is the time of the *j*th event for the *i*th neuron. Assuming a linear and time-invariant impulse response, the contribution to the net LFP by the *i*th neuron, *V_i_*(*t*), is simply the convolution of *s_i_* with either an AP or PSP waveform, *h*_AP/PSP_(*t*):

(3) $$V_{i}(t)=\int_{-\infty }^{\infty }s_{i}(\tau )h_{AP/PSP}(t-\tau )d\tau .$$

The net recorded LFP, *V_N_*(*t*), is then the sum of the contributions from all neurons:

(4) $$V_{N}(t)=\sum_{i=1}^{N}V_{i}(t).$$

Note that this simple model does not consider the effect of neuron location or complex electrode filtering on the recorded LFP waveform.

#### Synchronous constructed LFP model

We used the constructed model to determine the output of a network that is driven by a defined periodic input. This simulates a situation in which there is some physiological process driving all cells nearly synchronously at a certain frequency. This is similar to the pyramidal-interneuron gamma feedback loop in our biophysical model and others (Traub et al., 1997), but there are many physiological situations similar to this (e.g. theta rhythm, thalamocortical loops, etc). In effect, a large number of cells receive a similar input that influences their firing, similar to having a “master clock” in the system with some random variation in each cell’s firing. We use these conditions to compare the ability of synchronous APs versus synchronous PSPs to generate HFOs. The input was set to a specific frequency, and each cell responded to that input with some “jitter” to represent intercellular variability. The jitter was Gaussian distributed (N) for each cell, as defined by the SD *σ*_jitter._ The time of the *j*th event of neuron *i* was therefore given by the following:

(5) $$t_{i}^{j}=jT+N(0,\sigma_{\text{jitter}}^{2}),$$

where *T* determines the period of network oscillation. Population events remain periodic indefinitely, and the parameter *σ*_jitter_ determines the degree of event synchrony, with smaller values of *σ*_jitter_ implying greater synchrony (see Fig. 9*A* for a depiction of the effect of this parameter).

#### Asynchronous constructed LFP model

However, under physiological conditions there is not always a “master clock” driving the network at a given frequency. One might expect that, in the absence of any communication between cells, the population output would be purely random. However, several results from our biophysical model suggested that even uncoupled networks sometimes produce coherent oscillations when the pyramidal cells are firing at similar frequencies. To explore this unexpected result, we created another implementation of this LFP model to generate asynchronous network events. The goal of this model was to explore the emergence of HFOs from asynchronously spiking cells.

The underlying statistical procedure for generating event sequences assumed that: (1) the entire population of neurons had a mean event rate, but that there was variability in each cell’s specific rate (as well as in each cell’s initial phase), and b) each cell also exhibited variability, or “jitter”, from event to event. These sources of variability, as well as the independence of all event sequences, were important to investigate the possibility that asynchronous neuronal spiking might generate ripples and fast ripples. Formally, interevent jitter was modeled by assuming that given the *j*th event of neuron *i* occurs at time $t_{i}^{j}$, the next event $t_{i}^{j+1}$ will occur at some later time Gaussian-distributed about neuron *i*’s intrinsic interevent interval, *μ_i_*:

(6) $$p(t_{i}^{j+1}=t|\text{last}\;\text{event}\;\text{time}=t_{i}^{j})=N(t_{i}^{j}+\mu_{i},\sigma_{\text{jitter}}^{2}).$$

To account for population heterogeneity in intrinsic frequency, each μ*_i_* was drawn from $N(\mu_{\text{pop}},\sigma_{\mu }^{2}).$ The parameter *σ*_μ_ therefore quantifies how similar the intrinsic firing rates are among different cells, whereas *σ*_jitter_ quantifies how consistent an individual cell’s firing rate is. Note that without the presence of any form of coupling between the different “neurons” (i.e. event sequences) in the population, all neurons undergo events independently, resulting in completely asynchronous network dynamics. (Neuronal events were initialized with uniformly random values, so that the network began in an asynchronous state.) We studied the generation of ripples and fast ripples in this model while fixing the population mean μ_pop_ to be 5 ms (corresponding to a mean population frequency of 200 Hz). Simulations using AP waveforms featured 100 different cells, whereas those using IPSP waveforms featured 1500 different cells (reflecting the much larger number of synapses—in comparison with spiking compartments—that contribute to the LFP).

### Data Processing

All spectrograms were obtained using a sliding Gaussian window with a SD of 10 ms and a frequency resolution of 4 Hz. Fast ripples were defined to occur when the peak normalized power in the fast ripple band (>250 Hz) exceeded the peak normalized power in the ripple band (100–250 Hz).

The normalized power values depicted in Figures 9 and 10 were computed by first applying Thomson’s (1982) multitaper power spectral density estimate to a given waveform, and then determining the total power contained within 5 Hz of the nominal frequency *f*. Each square represents an average over 100 different realizations of the “synchronous constructed LFP model” described above.

## Results

### HFOs Generated by Input to either Basket Cells or Pyramidal Cells

**Figure 2:**
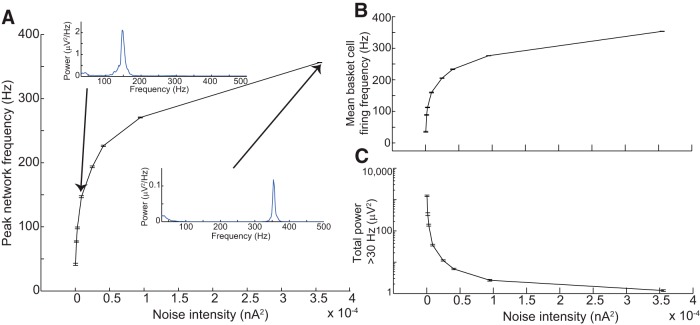
HFOs resulting from noisy input to the basket cell population. ***A***, Peak network frequency (defined as peak frequency of the LFP power spectral density) increased as the intensity of noisy synaptic input to basket cells increased. Insets, Two example PSD functions. Note the difference in scale between the vertical axes of the two insets, indicating the extreme diminution of oscillation amplitude as frequency increased. Individual PSDs were obtained from 1000 ms of simulation data. ***B***, Mean basket cell firing frequency very closely tracked peak network frequency for a given level of synaptic input. ***C***, Total LFP power >30 Hz decreased dramatically as noisy intensity (and peak network frequency) increased. Therefore, although it was possible for noisy input to basket cells to elicit rhythms with fast ripple frequencies, such rhythms exhibited very low amplitude. All error bars represent SEM over 10 simulations.

We first used the biophysical model to determine the parameters necessary to produce the full range of HFOs. We found that HFOs could be elicited through two distinct mechanisms: coherent firing of the 20 basket cells (which sent GABAergic connections to all 3080 pyramidal cells) or the 80 activated pyramidal cells (Fig. 1). In both cases, coherent LFP oscillations were generated by uncorrelated, noisy synaptic input to basket and/or pyramidal cells. Figure 2 shows the results of elevated input to the basket cell population. As depicted in Figure 2*A*, power spectral density (PSD) plots from the LFP were generally unimodal, and peak network frequency increased monotonically with increasing intensity of noisy synaptic input, spanning a range from gamma oscillations to fast ripples. A fast ripple was defined as a waveform in which the peak power >250 Hz exceeded peak power in the 100–250 Hz band. Peak network frequency (Fig. 2*A*) closely matched mean basket cell firing rate (Fig. 2*B*), indicating that the LFP resulted from IPSPs induced in pyramidal cells due to basket cell firing. Basket cell AP waveforms were present but contributed very little to the LFP, due to basket cells’ small size. Although the peak frequency did reach the fast ripple range, it is crucial to point out that the amplitude of network oscillations decreased substantially as peak network frequency increased (Fig. 2*C*). The total power was much higher in gamma (<100 Hz) frequencies, and reached very small levels >200 Hz (Fig. 2*C*). Such small-amplitude oscillations would be unlikely to resolve above background noise levels in a live recording.

**Figure 3:**
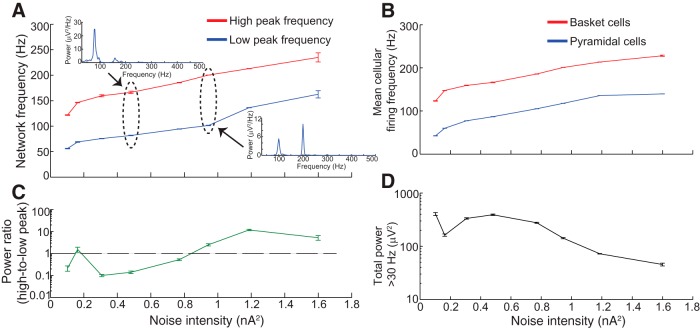
HFOs resulting from noisy input to pyramidal cell population. ***A***, Increased noise intensity to activated pyramidal cells stimulated increases in the two highest-power frequencies observed in the LFP PSDs, which were generally bimodal (see insets). Note that the network was incapable of generating rhythms faster than 250 Hz, in contrast with simulations in which basket cells received direct input (Fig. 2*A*). ***B***, High-frequency and low-frequency spectral bumps corresponded to the mean firing rates of basket cells and pyramidal cells, respectively. ***C***, Relative dominance between the two spectral bumps varied with noise intensity, as shown in this plot of the ratio of the maximum power of the high-frequency peak to the maximum power of the low-frequency peak. Ratios >1 (demarcated by black horizontal line) imply that the high-frequency peak dominated the low-frequency peak. ***D***, Total LFP power >30 Hz tended to decrease somewhat with increasing noise intensity, though not nearly as dramatically as when basket cells received noisy input (Fig. 2*D*). All error bars represent SEM over 10 simulations. Note that the pyramidal cells in this figure require higher levels of noise input to fire than the basket cells in Figure 2, as a result of their different input impedance. This disparity was also recently shown experimentally (Karlócai et al., 2014).

Network activity was distinctly different when noisy input was delivered to pyramidal cells rather than basket cells (Fig. 3*A*). The spectral content was bimodal due to the different firing rates of these two classes of cells. The lower frequency peak was due to pyramidal cell firing (Fig. 3*A*,B, compare the lower lines), which dominated the LFP at low noise intensity (Fig. 3*C*), and the higher-frequency peak was due to basket cell firing (Fig. 3*A*,B, compare the upper lines), which dominated the output for high noise intensities. Thus, when the pyramidal cells were driven by varying levels of synaptic activity, they produced a range of strong oscillations from 60–250 Hz. It is important to note that no level of noise intensity was capable of eliciting a network rhythm faster than about 250 Hz. The pyramidal cells reached firing rates of over 100 Hz, a level that would only be expected in highly active conditions such as epilepsy (see Discussion). Thus, this model shows the transition from what are likely normal to epileptic HFOs. However, with this configuration (all connections intact) it was impossible to elicit fast ripples for two reasons: (1) the basket cell inhibition effectively limited the peak frequency of pyramidal cell firing, and (2) pyramidal cells were synchronized when inhibitory feedback was intact.

### Peak Frequency Insufficient to Disambiguate Ripple Mechanisms

**Figure 4:**
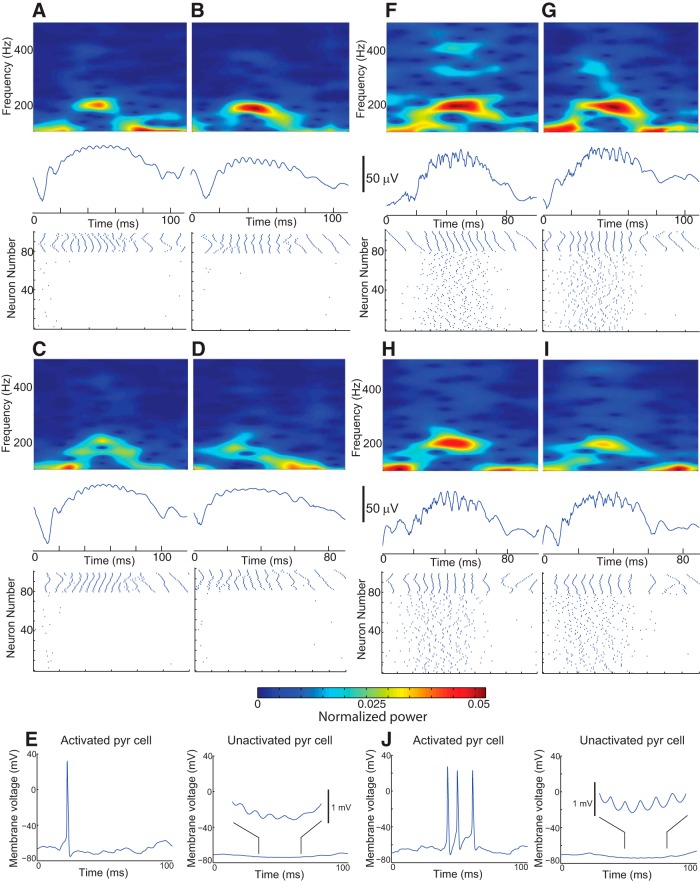
Ripple generation by noisy stimulation of either basket cells or pyramidal cells. ***A***–***D***, Examples of sharp-wave ripples induced by activation of the basket cell population (ID numbers 81–100 in the raster plot) with a noise intensity of 2.5 × 10^–4^ nA^2^. Note the sparsity of spiking of pyramidal cells (ID numbers 1–80 in the raster plot). LFP ripple oscillations were produced primarily by IPSPs in all 3080 pyramidal cells. (3000 of the pyramidal cells never fired and are not included in the raster plots.) ***E***, Example intracellular voltage traces of an activated pyramidal cell (left) and an unactivated pyramidal cell (right) resulting from activation of the basket cell population. Inset, IPSP-induced membrane oscillations in the unactivated pyramidal cell. ***F***–***I***, Examples of sharp-wave ripples induced by noisy synaptic stimulation of the activated pyramidal cell population (with a noise intensity of 0.77 nA^2^). The increased pyramidal cell spiking induced increased activity of basket cells, whose inhibitory influence restricted the dominant frequency component to ≈200 Hz. ***J***, Example intracellular voltage traces of an activated pyramidal cell (left) and an unactivated pyramidal cell (right) resulting from noisy synaptic stimulation of the activated pyramidal cell population. Inset, IPSP-induced membrane oscillations in the unactivated pyramidal cell.

HFOs have traditionally been categorized based upon peak frequency into fast gamma, ripples, and fast ripples. However, as shown in Figures 2 and 3, our biophysical model was capable of generating ripples through both normal and epileptic conditions, similar to recent experimental work (Aivar et al., 2014). This leads to the question of whether ripples produced by these disparate mechanisms can be distinguished. To demonstrate the similarities, we depict example waveforms, spectrograms, and raster plots for simulated sharp-wave ripples elicited by these two different mechanisms (Fig. 4). In Figure 4*A*–*D*, we show examples of approximately 200 Hz rhythms elicited by elevated basket cell activation (noise intensity = 0.25 × 10^–4^nA^2^). These LFP rhythms reflect synchronous IPSPs induced in pyramidal cells, consistent with previous experimental studies (Buzsaki et al., 1992; Ylinen et al., 1995; Schlingloff et al., 2014). The sparse firing of pyramidal cells in these examples contrasts sharply with the alternative scenario (Fig. 4*F*–*I*) in which pyramidal cells, rather than basket cells, were directly activated (noise intensity = 0.77 nA^2^).

Figure 4*F*–*I* shows that the peak frequency was again around 200 Hz, but network activity was dramatically different: the elevated activity of pyramidal cells induced increased basket cell firing, so that pyramidal cell spiking and basket cell-induced IPSPs contributed fairly equally to the LFP oscillations. Although peak frequency is similar in both situations, the spectrograms show that there are other differences between the two cases: there was more high-frequency activity with AP-generated ripples (see increased 300–400 Hz power in spectrograms Fig. 4*F*–*I*), whereas the IPSP signals (*A*–*D*) were smoother, without high-frequency content. These examples illustrate the importance of evaluating more than simply the peak frequency when attempting to discern the underlying cause or pathogenicity of HFOs.

### Effects of Compromised Inhibition

**Figure 5:**
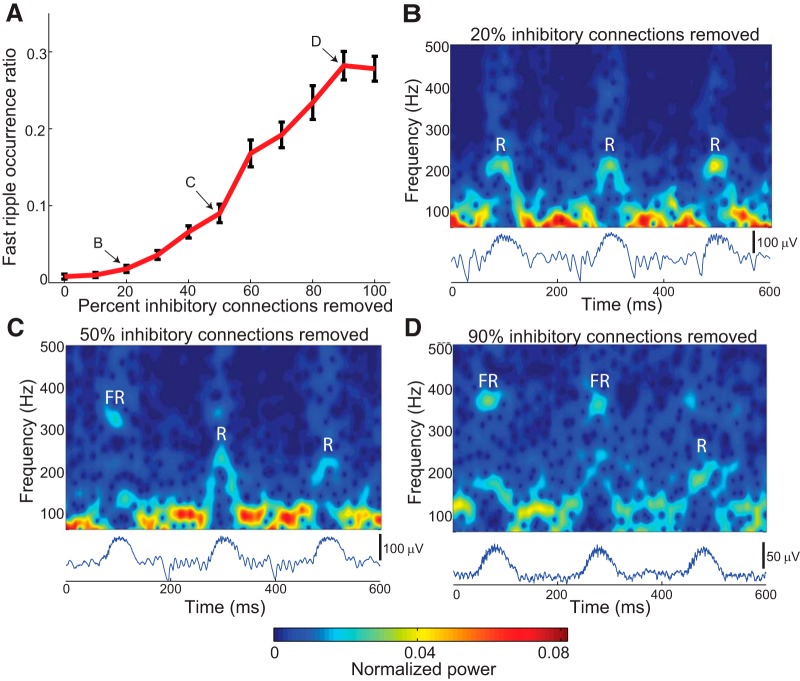
Effect of diminished inhibition on fast ripple incidence. Simulations were conducted in which 50 separate sharp waves were induced by intermittently activating pyramidal cells with noisy input (using a noise intensity of 0.77 nA^2^). Inhibitory connections from basket cells to all pyramidal cells were progressively removed. A fast ripple was defined to occur when the peak energy in the fast ripple band (>250 Hz) exceeded the peak energy in the ripple band (100–250 Hz). ***A***, Proportion of sharp waves which exhibited fast ripples, as a function of percent inhibitory connections removed. Fast ripple incidence increased dramatically as inhibitory connections were lost. (Error bars represent SEM over 10 simulations, each with 50 induced sharp waves.) ***B***–***D***, Example LFP’s and spectrograms for three levels of intact inhibition, each with three example sharp waves. FR, Fast ripple episode; R, ripple episode.

Because the model network was unable to generate fast ripples for any level of input when network inhibition was intact (Fig. 3), we investigated the effects of compromising network inhibition on fast ripple generation. Starting from the model shown in Figure 3 with a noise intensity of 0.77 nA^2^, we randomly disabled a specified percentage of basket-to-pyramidal cell GABAergic connections, then generated 50 consecutive sharp waves by transiently increasing the random synaptic input to pyramidal cells. This enabled determination of the proportion of sharp waves that included fast ripple events. (The peak frequency in the fast ripple band had to have higher spectral power than the peak frequency in the ripple band for at least 25% of the duration of the sharp wave in order to count as a fast ripple event.) From the results shown in Figure 5, disruption of inhibitory connections had a profound impact on the emergence of fast ripples: as more basket cell connections were lost, the same input that had previously generated only sharp wave-ripple events began to produce fast ripples as well. This result is similar to previous findings that decreased GABAergic transmission facilitates the emergence of fast ripples (Bragin et al., 2002a; Demont-Guignard et al., 2012).

**Figure 6:**
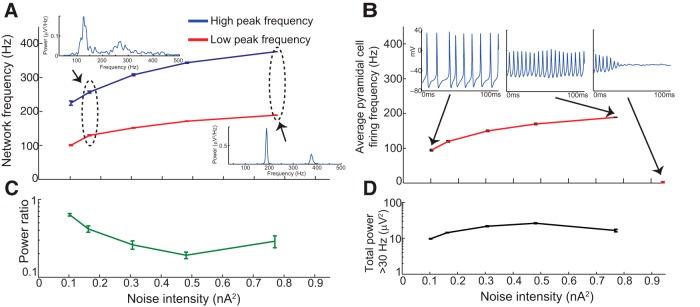
HFOs resulting from noisy input to 80 uncoupled pyramidal cells. ***A***, Two highest-power frequency peaks in the PSD as a function of noise intensity. PSDs were generally bimodal, and grew more coherent as noise intensity increased. Note how the high-frequency peaks reached fast ripple frequencies and represent a harmonic of the low-frequency peaks. ***B***, The low-frequency peak in the PSDs from ***A*** corresponded to the average cellular firing frequency. Insets, Representative voltage traces of individual neurons for three different levels of noise intensity. The right column shows how the entire network went into depolarization block when stimulated with high enough noise intensity. ***C***, Ratio of peak power of the high-frequency peak-to-peak power of the lower-frequency peak. ***D***, Total LFP power >30 Hz as a function of noise intensity. As in Figure 3, there were coherent oscillations even at high noise intensities. All error bars represent SEM over 10 simulations.

Loss of network inhibition has two important consequences in our model. First, fewer inhibitory connections imply a relatively greater contribution to the LFP from pyramidal cell APs compared with IPSPs. Second, as more feedback inhibition is lost the network progressively loses its ability to synchronize, until at extreme levels, it is completely uncoupled and all pyramidal cells are independent and asynchronous. Yet, as shown in Figure 5*D* (90% of inhibitory connections removed), coherent HFOs arise even with most inhibition is removed, allowing most cells to fire asynchronously. Similar results were found with 100% of feedback inhibition removed (Fig. 6*A*). These observations raise two questions: (1) how does asynchronous neuronal spiking generate ripples and fast ripples, and (2) to what degree are AP-dominated versus PSP-dominated LFPs capable of producing fast ripples?

### Generation of Ripples and Fast Ripples from Asynchronous Network Activity

To address the first question, we analyzed the same network from Figure 3 (activation of pyramidal cells), but removed all inhibitory basket cell connections. There was no coupling of any sort between any pyramidal cells in the network, which allowed them to fire completely independently. Figure 6*A* shows that this completely asynchronous network generated coherent network oscillations characterized by a pronounced fundamental frequency and a lower-power first harmonic. This is somewhat similar to the bimodal PSDs observed when pyramidal cells received input when feedback inhibition was intact (Fig. 3*A*), except that the frequencies are higher, and importantly, there were no individual cells that fired at the frequency of the second harmonic (Fig. 6*B*). Note that the second harmonic constitutes a fast ripple frequency and that unlike the situation in which basket cells were directly activated (Fig. 2*C*); in this case, the oscillation amplitude does not appreciably diminish with increasing noise intensity. Instead, total power remained roughly constant as noise intensity increased (Fig. 6*D*), until the entire network went into depolarization block and all oscillations ceased (Fig. 6*B*).

**Figure 7:**
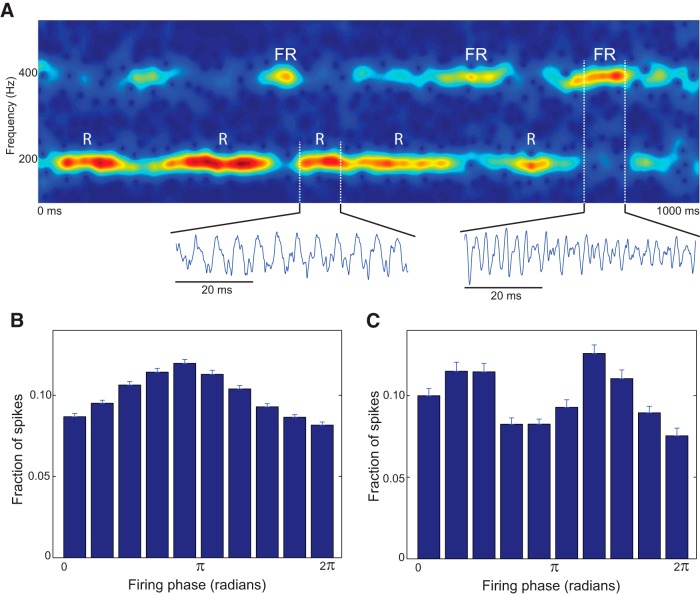
Emergence of fast ripples in an uncoupled, asynchronously spiking network. Using the same parameters as the highest-frequency data in Figure 6, a 20,000 ms simulation was performed to identify the emergence of HFOs in a network of 80 uncoupled pyramidal cells driven by high levels of uncorrelated noisy input. ***A***, LFP spectrogram of a 1000 ms interval demonstrates that both ripple (R) and fast ripple (FR) episodes emerged sporadically. ***B***, Spike-timing histogram relative to ripple phase, averaged over all 78 observed ripple episodes. ***C***, Spike-timing histogram relative to ripple phase, averaged over all 26 observed fast ripple episodes. Ripples occurred when the 80 cells achieved brief unimodal spike time distribution, and fast ripples occurred when the distribution was transiently bimodal. Error bars represent SEM over all observed ripple/fast ripple episodes.

Figure 6*A* shows that the ripple (<250 Hz) frequencies dominated despite the presence of the fast ripple harmonics. However, our observations of the raw data revealed that there were many instances in which fast ripples dominated, just as in Figure 5*C*,D. To investigate how such fast ripple activity emerges in this asynchronous network, we ran a long simulation (20,000 ms; Fig. 7) using the same uncoupled pyramidal cell network as used in Figure 6, with the noise intensity set to the highest level that sustained network oscillations without going into depolarization block (0.77 nA^2^). As shown in Figure 7*A*, we observed that strong ripple oscillations at ≈200 Hz (which matched the mean firing rate of individual neurons) dominated the LFP the majority of the time, but that fast ripple episodes emerged spontaneously, typically lasting 20–50 ms. Spike-time histograms relative to ripple phase indicated that the ripple episodes had a single cluster (Fig. 7*B*), whereas fast ripple episodes occurred due to two out-of-phase spiking clusters (Fig. 7*C*). This is similar to what has been proposed previously by Menendez de la Prida’s group (Foffani et al., 2007; Ibarz et al., 2010). Most striking, however, is that in our results there is no organizing mechanism for such bicluster dynamical states; they emerge briefly and spontaneously from asynchronous activity in a completely uncoupled network. Such fast ripples are therefore not a result of decreased spike timing reliability, but emerge by chance when the randomly evolving spike-time structure happens to assume a bimodal form.

### Constructed LFP

It is somewhat counterintuitive that an uncoupled network could produce coherent oscillations, and given the complexity of the biophysical model, we wanted to exclude the possibility that such coherence was an artifact of the simulation itself. We therefore developed a simplified model stripped of all biophysical details, in which LFP signals were “constructed” by convolving action potential waveforms with a number of randomly generated spike trains, each representing the firing times of a single cell. This model assumed the existence of a network drive for cells to fire near a given frequency, but with two primary sources of variability in the spiking of each cell. This model contained no physiological mechanisms that dictated how and when APs or PSPs appeared, and instead simply showed how the LFP would appear if such activity occurred at a given time.

**Figure 8 F8:**
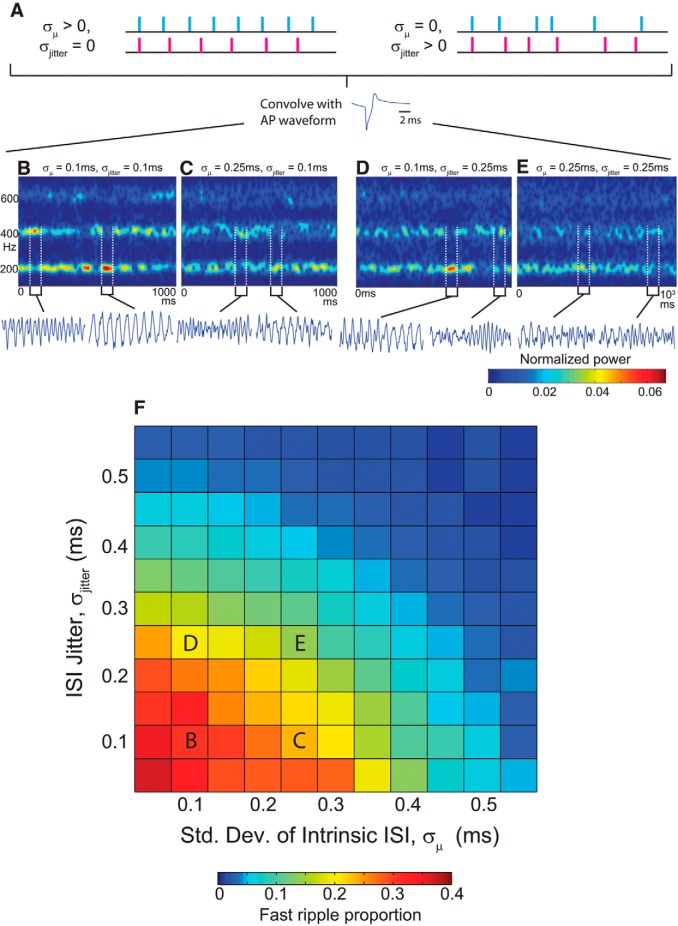
Fast ripple occurrence ratio for constructed LFP’s generated from AP waveforms. ***A***, Constructed LFPs were produced by first generating trains of event times which had two sources of variation: (1) different trains had different values for their intrinsic firing rates, with greater values of *σ*_μ_ implying greater firing rate heterogeneity between different cells, and (2) within each train there was “jitter” in the ISI. These spike trains were then convolved with AP waveforms and summed to yield the constructed LFP. Red and blue spike trains show examples of firing times of two different “cells” with different firing rates (left) or different ISI jitter (right). ***B***–***E***, Spectrograms and LFP samples from the constructed LFPs generated using the indicated parameters. Fast ripples frequently emerged from random network firing. ***F***, Fast ripple proportion as a function of the two sources of heterogeneity in the model. Fast ripple proportion was simply the proportion of total time that the peak frequency in the 100–700 Hz band was >250 Hz.

The constructed LFP model assumed that there were many cells firing near a given frequency due to conditions in the network. This is similar to the exploration of the dependence of network-preferred HFO frequency on intrinsic firing rates of individual cells, as explored in Ibarz et al. (2010). Although each cell was driven in similar fashion, there were two primary sources of variability in spike times. The first was motivated by the fact that in the brain, each cell generally has different parameters and inputs, and thus each will have slightly different mean firing rates for a given brain state. We modeled each cell’s mean interspike interval (ISI) μ*_i_* as being drawn from a normal distribution with standard deviation *σ*_μ_. The second source of variability modeled “jitter” in ISI times, since each cell’s ISI typically fluctuates from spike to spike due to noise in the network, even when the mean firing rate is relatively constant over time. The degree of ISI jitter was determined by the parameter *σ*_jitter_. The difference in the effects of these two parameters is depicted in Figure 8*A*. (See Models, Asynchronous Constructed LFP Model for further details.)

#### Coherent ripples and fast ripples from an asynchronously spiking network

We first used this model to explore the potential for asynchronous AP-dominated HFOs, as suggested in the biophysical model (Fig. 7*A*). Figure 8*B*–*E* demonstrate that in a generic model of network activity stripped of all biophysical details, asynchronous neuronal activity can produce strong LFP oscillations. As in the biophysical model, this constructed model displayed a prominent oscillation at the overall mean cellular firing frequency (200 Hz, corresponding to μ = 5 ms), intermixed with transient fast ripple episodes. As heterogeneity in mean ISI or ISI jitter increased, the LFP became noisier, LFP oscillations less coherent, and fast ripple episodes less frequent. Figure 8*F* shows the fast ripple occurrence ratio as a function of both sources of dynamical heterogeneity, which had very similar overall effect. In both cases, fast ripple occurrence decreased as heterogeneity increased, with heterogeneity of ∼10% of mean ISI effectively eliminating fast ripple episodes.

#### Generation of fast ripples by APs versus PSPs in a synchronous network

**Figure 9 F9:**
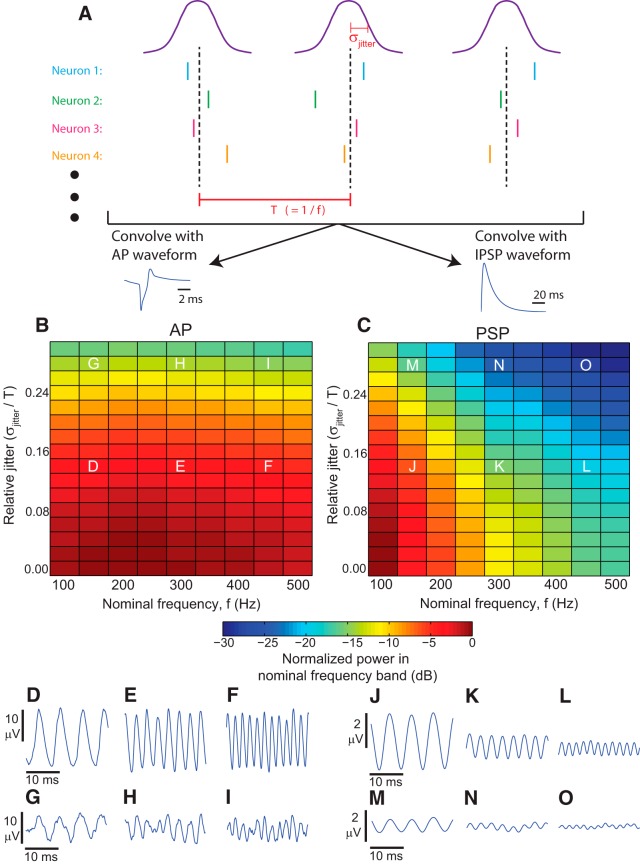
Capability of AP versus PSP events to produce HFOs of varying frequency. ***A***, Constructed LFPs were produced by generating synchronous network bursts of either AP or PSP events at periodic intervals, with nominal frequency *f* and random variation defined by *σ*_jitter._ ***B***, ***C***, Color encodes the power within the frequency band *f* ± 5 Hz, normalized by the maximum power observed across all parameters in each waveform. For both APs and PSPs, increased jitter caused the LFP output to become less coherent and the normalized power to drop. On the other hand, increasing frequency of network bursts had little effect on AP-dominated oscillations, but resulted in significant attenuation of PSP-dominated oscillations. ***D***–***I***, Representative AP-dominated LFPs associated with the corresponding combinations of parameters indicated in ***B***. Note how amplitude is unchanged as oscillation frequency increases. ***J***–***O***, Representative PSP-dominated LFPs associated with the corresponding combinations of parameters indicated in ***C***. Note the difference in scale bars, and how amplitude is dramatically attenuated as oscillation frequency increases. Thus, APs can robustly produce the full range of HFOs, whereas PSPs are unlikely to produce HFOs >200 Hz.

We then used this construct to test another important aspect of HFOs: the differing features of HFOs caused by APs versus IPSPs. Experimentally, both IPSPs (Ylinen et al., 1995; Klausberger et al., 2003b; Spampanato and Mody, 2007; Schevon et al., 2009) and APs (Csicsvari et al., 2000; Grenier et al., 2003; Jiruska et al., 2010; Bragin et al., 2011) have been shown to contribute to ripples, a phenomenon that was replicated by our biophysical model (Fig. 4*A*–*D* shows IPSP-dominated ripples, whereas *E*–*H* show AP-dominated ripples). Less is known, however, about these two waveforms’ respective abilities to generate fast ripples. Our constructed LFP model is an ideal method for investigating this question. We generated event times in a similar manner to that depicted in Figure 8*A*, except that events were clustered in synchronous network bursts, with the parameter *σ*_jitter_ determining the degree of synchronization within each burst (Fig. 9*A*). Event times were then convolved with either IPSP or AP waveforms to yield the LFP (see Models, Synchronous Constructed LFP Model), so overall this approach modeled the rhythm resulting from a periodic drive to the network. By increasing the nominal frequency of the periodic drive, we were able to investigate the degree to which IPSP versus AP waveforms could generate robust rhythms at various frequencies, from ripples to fast ripples.

As shown in Figure 9*D*–*O*, both AP-dominated and IPSP-dominated LFPs exhibited coherent oscillations whose dominant frequency matched the nominal network burst frequency (though AP-dominated LFPs grew less “clean” as coherence decreased, as shown in Fig. 9*D*,*G*). We explored the ability of both classes of waveforms to generate fast ripples by observing how LFP oscillation amplitude was affected by increased frequency of network bursts. The color plots in Figure 9*B*,*C* show that AP-dominated and IPSP-dominated LFPs exhibited very different trends: the amplitude of AP-dominated LFPs remained constant as frequency increased, whereas the amplitude of IPSP-dominated LFPs decreased dramatically with increasing frequency. These trends are even more starkly depicted in the plots of LFP waveforms shown in Figure 9*D*–*O*. Furthermore, decoherence of network bursts (resulting from increased *σ*_jitter)_ had essentially the same impact upon AP-dominated LFP amplitude across all frequencies (Fig. 9*B*). The impact of decoherence upon IPSP-dominated LFPs, on the other hand, grew more severe as frequency increased (Fig. 9*C*).

These results show that, independent of the underlying network structure, the actual waveforms that arise when a population of cells produces either APs or IPSPs have extremely different capacities to produce fast ripples. The short duration of APs allows a wide range of frequencies that are resistant to significant jitter among the cells. In contrast, although IPSPs are theoretically able to generate fast ripple signals, the amplitude is extremely low and even small amounts of jitter abolish the signal. We conclude that, under physiological conditions, it is likely that all fast ripples are generated purely by APs, regardless of underlying network structure.

#### Effect of synaptic parameters on fast ripple generation by PSPs

**Figure 10 F10:**
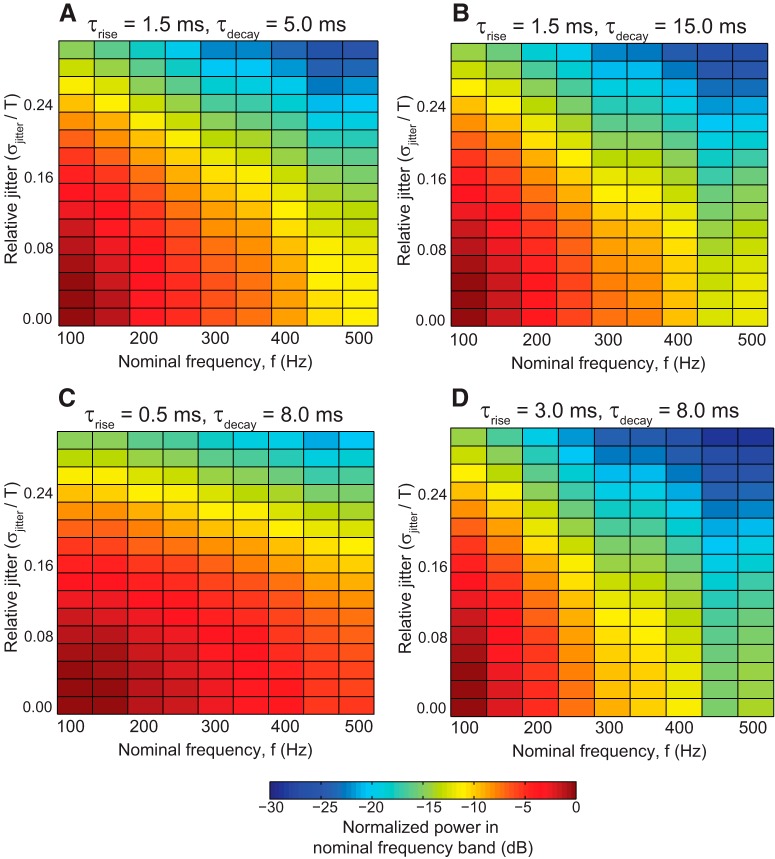
Effects of synaptic parameters on HFOs. LFPs were constructed as in Figure 9*C*, except that GABAergic synaptic rise and decay times were modified from their standard values (*τ*_rise_ = 1.5 ms; *τ*_decay_ = 8.0 ms). ***A***, ***B***, Changing *τ*_decay_ had little effect on the HFO output. ***C***, ***D***, Very fast *τ*_rise_ time (0.5 ms) enabled IPSPs to produce HFOs more robustly.

Previous work has shown that network rhythms are dramatically affected by changes in synaptic parameters, with faster time constants (such as synaptic rise time) having a much greater impact than slower time constants (such as synaptic decay time; Brunel and Wang, 2003). We investigated the effects of varying these synaptic parameters in our constructed LFP model, with the results shown in Figure 10. For all modifications (increasing and decreasing *τ*_rise_ and *τ*_decay_), the amplitude of PSP-dominated LFPs decreased with increasing frequency, as with the standard synaptic parameters. Modifying *τ*_decay_ had very little effect on network rhythms (compare Figs. 10*A*,*B* with 9*C*), whereas decreasing *τ*_rise_ did result in more robust network rhythms at high frequency (Fig. 10*C*). With supraphysiologically fast rise time (0.5 ms), the output approached the results of APs (Fig. 9*B*), but was still less robust at higher frequencies. Both of these results are consistent with the findings of Brunel and Wang (2003). It should be emphasized, however, that in all cases increasing frequency resulted in greater sensitivity to decoherence (i.e., increased relative jitter), an effect that was not observed in AP-dominated LFPs (Fig. 9*B*). These results show that due to their slower dynamics, PSPs are unlikely to produce fast ripples even with different synaptic parameters.

In this simplified model, therefore, PSP-dominated LFPs were in principle capable of producing fast ripples, but these rhythms were much less robust than the fast ripples generated by APs. They required extreme network coherence and produced very small amplitude signals, and would therefore be unlikely to be observed in networks with physiological levels of noise.

## Discussion

This work explores the unifying, generic phenomena necessary to produce HFOs, independent of any specific network structure. Within this framework, we find that normal ripples, pathological ripples, and fast ripples can be formed on a single continuum within the same model while a wide range of synaptic activity is presented to the cells. Our model leads to several important predictions. First, peak frequency is insufficient to determine when ripple HFOs (100–250 Hz) are pathological, as both AP-based and IPSP-based mechanisms can produce similar activity. Second, fast ripples (>250 Hz) can be produced by a wide range of different parameters. Third, identical parameters can produce either pathological ripples or fast ripples at different times, similar to experimental data. Fourth, AP-based HFOs can arise from completely asynchronous networks, requiring no specific connectivity or organization at all. These findings suggest that HFOs are an inherent behavior of networks of similar pyramidal cells: any situation that permits cells to fire at high frequency and with similar rates will produce “pathological” HFOs, potentially even under normal conditions that have similar parameters.

### Choice of Models and Parameters

This work has utilized two models, a biophysical model to show how network interactions produce different HFOs, and a constructed LFP model to show the differences between HFOs produced by IPSPs and APs. The biophysical model contains a simple hippocampal network, which assures that we are simulating generic, rather than structure-specific, phenomena. Because it was designed to simulate fast oscillations, it did not include the slower effects of OLM cells that produced theta oscillations in the original presentation of this model (Tort et al., 2007). Omission of OLM cells (and several other potential interneurons) does eliminate some effects that might be important in generation of particular HFOs. For instance, recent work has suggested OLM cells are involved in some HFOs (Varga et al., 2012; Pangalos et al., 2013), although other work showed that OLM cells were silent during HFOs (Klausberger et al., 2003a). More complex HFO models show the effects of several other interneurons in producing “normal” ripples (Schomburg et al., 2012), or complex networks of axo-axonal gap junctions producing fast ripples (Simon et al., 2014). These models, and the others we have previously discussed, contain some effects not present in our model, which are likely to produce subtle differences in the HFO characteristics; however, these are not generic mechanisms of HFO generation, and it is difficult to compare the results between such models. Our goal with the current model was to investigate the unifying mechanisms of HFOs, from gamma to fast ripples, which might reconcile such different networks.

One critical parameter in our biophysical model is the intensity of synaptic noise, which is the primary force driving cellular activity. It is important to determine whether the range of noise intensity in our model is physiologically plausible. The lower levels of noise intensity are easily justified, but several of the effects in this work arise only when the noise reaches extremely high levels. For instance, we demonstrate ripples produced by either basket cells or pyramidal cells firing at 200 Hz (Figs. 2, 6). In the interneurons, whereas some studies have shown that basket cells do not reach this frequency under certain conditions (Massi et al., 2012; Povysheva et al., 2013), others have validated that they can reach these firing rates during HFOs (Klausberger et al., 2003a; Hájos et al., 2013). Similarly, it is uncommon for pyramidal cells to fire so fast, and some of our results show cells driven into depolarization block (Fig. 6*A*,*B*). Although such a high level of noise intensity may seem extreme, it is actually common during epileptic conditions (Grenier et al., 2003; Dzhala and Staley, 2004; Stacey et al., 2009; Jirsa et al., 2014; Karlócai et al., 2014) and not unlike other physiological conditions, such as the Up state (Destexhe et al., 2003). This powerful input is the key to our model of HFOs in uncoupled cells. Because neighboring pyramidal cells have similar structural and dynamic parameters, their absolute refractory periods and peak firing rates are also similar. Thus, when there is a strong enough network drive, cells will have similar firing rates, which can produce coherent oscillations regardless of network connectivity. A recent study by Alvarado-Rojas et al. (2014) provides strong experimental support for this scenario.

We developed the constructed LFP model to answer several questions regarding how HFOs can be formed by APs versus IPSPs. This model gave us explicit control of when the events occurred, allowing us to investigate how the LFP would appear under a vast range of different network firing, independent of the specific mechanisms that would produce such firing. It is important to point out that each waveform template (i.e., the signal produced when an AP or IPSP occurred) was actually recorded from the biophysical model. Thus, although there was no neuronal network in this model, its output approximates any implementation of the biophysical network that produced the same firing times. Note, however, that this model does not take into account the nonlinear spatiotemporal relationships between different somatodendritic sinks and sources that comprise LFP signals.

### Mechanisms of Normal and Pathological HFOs

It is currently thought that normal ripples are produced either nearly exclusively by IPSPs (Ylinen et al., 1995; Le Van Quyen et al., 2008) or a roughly equal mixture of IPSPs and active currents (Schomburg et al., 2012), whereas epileptic HFOs are most likely produced predominantly by the active currents associated with population spikes (Bragin et al., 2011). Our biophysical model is consistent with this view, because noisy synaptic bombardment of either basket cells (which generated an IPSP-dominated LFP) or pyramidal cells (which generated an LFP comprised of both APs and IPSPs) tended to produce ripples when inhibition was intact (Fig. 4), and abnormal ripples when inhibition was compromised (Fig. 5). The mechanisms underlying fast ripple generation have been more challenging to explain. Previous studies on pathological HFOs (Foffani et al., 2007; Demont-Guignard et al., 2012; Wendling et al., 2012; Aivar et al., 2014; Karlócai et al., 2014) have shown that fast ripples occur when pyramidal cells become very excitable and inhibition is compromised. Many specific mechanisms have been investigated both experimentally and computationally: axo-axonal gap junctions (Traub and Bibbig, 2000; Schmitz et al., 2001; Traub et al., 2005; Roopun et al., 2010; Simon et al., 2014), recurrent synapses (Dzhala and Staley, 2004; Ibarz et al., 2010), spike time variability (Foffani et al., 2007), uncorrelated firing (Demont-Guignard et al., 2012), decreased Ca^2+^ concentration (Aivar et al., 2014), and disconnected populations (Ibarz et al., 2010). All have demonstrated some experimental evidence, and each may exist under different conditions, but reconciling these theories has been controversial.

In this study, we take an alternative approach by focusing on the general dynamical properties of network activity necessary to generate HFOs, rather than specific lower-level mechanisms. We provide a generic framework to identify and unify the mechanisms underpinning normal ripples, pathological ripples, and fast ripples in the hippocampus. In general, normal gamma and HFOs arise when low to moderate levels of network drive induce coherent IPSP firing. As the drive increases, pathological HFOs arise when pyramidal cells become highly active, under any particular network structure (Fig. 4). At ripple frequencies, the output can be any combination of IPSP and AP waveforms. High levels of inhibitory feedback in the network are likely to limit pathological HFOs to ripple frequencies (Fig. 3). However, as the pyramidal cells become very active, it becomes more and more likely that they will transiently desynchronize if inhibition is impaired (Fig. 5). This transition from normal to epileptiform activity as noise increases is reminiscent of recent work showing how a network moves from the normal to seizure regime (Jirsa et al., 2014). Like that work, our model shows that the same network activity can result from a variety of underlying mechanisms.

#### Asynchronous HFOs

Although several previous studies have assumed that AP-dominated ripples result from highly synchronous pyramidal cell firing (Bragin et al., 2010; Menendez de la Prida and Trevelyan, 2011), our results suggest an alternative scenario is also possible: that AP-dominated ripples in fact do not require any specific structure at all. They may result from asynchronous firing of a population of pyramidal cells driven near their maximum firing rate. Figures 7 and 8 illustrate how a population of independently and randomly firing pyramidal cells may generate a strong ripple oscillation when each fire at ≈200 Hz (which several studies have shown to be a realistic rate under pathological conditions; Grenier et al., 2003; Dzhala and Staley, 2004; Aivar et al., 2014; Karlócai et al., 2014). This result is consistent with previous theoretical studies showing that asynchronous neuronal firing can produce coherent LFP rhythms (Ray et al., 2008; Nunez, 1995).

#### Emergent fast ripples

Fast ripples will in turn transiently and sporadically emerge from such pathological ripples (Figs. 5, 7, and 8) provided there are enough spikes to produce an LFP signal, since fast ripples almost certainly must be comprised of APs (Fig. 9). Fast ripples thus do not depend upon a specific network structure, but are a general, emergent phenomenon. The only requirements to produce fast ripples are that: (1) pyramidal cells are very active, (2) the cells can become desynchronized, and (3) the LFP is dominated by APs. We predict that any network conditions that produce these effects will be capable of generating fast ripples. Note that these conditions do not necessarily imply pathological activity, which may explain why fast ripples have been observed in normal cortex (Amassian and Stewart, 2002; Blanco et al., 2011; Kucewicz et al., 2014).

#### Comparison with past models

Fast ripples emerge in our model when two clusters of cells fire out of phase (Fig. 7*C*). This is similar to past work (Foffani et al., 2007; Ibarz et al., 2010) but with a crucial difference: in our model the cells can be completely uncoupled. This results in completely asynchronous activity producing ripples which briefly form fast ripples when the population assumes a bimodal state (Figs. 7, 8*B*–*E*). For a ripple rhythm generated by asynchronous spiking, increased jitter will impair the emergence of fast ripples (Fig. 8*F*). In contrast, for ripples generated by synchronous firing (Foffani et al., 2007) increased jitter will facilitate fast ripple formation. These two mechanisms therefore approach fast ripple activity from opposite ends of the spectrum: one starts with highly synchronous activity, the other with completely asynchronous activity, so that different network conditions induce different paths to fast ripple generation. The possibility that initially asynchronous activity might generate pathological rhythms is suggested by experimental evidence of asynchronous firing in interictal discharges (Alvarado-Rojas et al., 2013) and epileptic seizures (Truccolo et al., 2011), as well as previous work comparing epileptic spikes and fast ripples which found a clear role for asynchronous firing (Demont-Guignard et al., 2012). Two novel features of our model are: (1) its ability to describe the transition from normal gamma to abnormal fast ripples in a single set of parameters, and (2) it provides a mechanistic link between pathological ripples and fast ripples, thereby helping to explain why fast ripples are often intermixed with ripples (Bragin et al., 1999; Worrell et al., 2008), as well as why fast ripples are so ephemeral.

### Features of Normal and Pathological HFOs

Even after nearly two decades of research, there is still no clear way to determine whether an HFO is produced by normal or abnormal mechanisms. What is clear is that peak frequency alone is insufficient to make the distinction. Less than 250 Hz, HFOs can be indicative of either completely normal or epileptic activity. Fast ripples originally appeared to be more specific to epilepsy in hippocampus, but recent human data have placed that in doubt as well (Kucewicz et al., 2014), and fast ripples have been well known in normal somatosensory cortex for many years (Amassian and Stewart, 2002; Baker et al., 2003; Barth, 2003; Blanco et al., 2011). Thus, additional methods are needed to distinguish normal from abnormal HFOs.

Our data suggest two important aspects of abnormal HFOs that may help in future research. First, the fact that fast ripples emerge from pathological ripples may explain why they are transient and coexist with ripples on the same electrode recordings. This suggests an alternative strategy of searching for similarities between such events as harmonic frequencies or other features, rather than assuming they are different. Second, our biophysical simulation (Fig. 4) demonstrates that although peak frequency may be similar in normal and epileptic HFOs, there are more subtle features of the signal such as high frequency band power (>250 Hz) that might distinguish them. The rigorous solution to this question will require large amounts of human data in which vast numbers of HFOs can be analyzed. Recent work using controlled stimulation has shown that different HFOs can be distinguished using basic features (Matsumoto et al., 2013; Kucewicz et al., 2014); our results can help guide future analysis of such signals to find more comprehensive differences.

## Conclusion

Distinguishing normal from pathological HFOs remains a challenging problem whose solution holds great promise for people with epilepsy. In this study, we have focused on the network mechanisms that differentiate the varieties of HFOs, motivating future experimental studies to obtain a more comprehensive picture of network activity. This study also provides a foundation for investigating differential LFP signatures for normal versus pathological HFOs, and guides future experimental and clinical HFO research.

## Synthesis

The decision was a result of the Reviewing Editor Liset Menendez de la Prida and the peer reviewers coming together and discussing their recommendations until a consensus was reached. A fact-based synthesis statement explaining their decision and outlining what is needed to prepare a revision is listed below. The following reviewer agreed to reveal his identity: Jiannis Taxidis

### Editor's synthesis:

A note on reviewers' identity of this version: R1 here was R2 of the previous version. R2 here is a postdoc of R1 of the previous version, who also participated in generating the report. Hence your ms was evaluated by the same reviewers and me.

Final decision: Revise and re-review. Both reviewers recognize the manuscript may advance the field yielding some interesting new insights on the generic processes underlying normal and abnormal forms of hippocampal high-frequency oscillations. While authors have addressed most of our concerns for the previously rejected submission (requiring more than 2 months' work) and the ms is substantially improved, there are still several remaining. I therefore would reconsider a revised version.

In particular, major points that still require some work are:

1) The ms is lengthy and poorly concise. It would benefit from some focus and emphasis on novelty. Regarding HFOs, the merit of the article is to conceptualize in generic terms the basic processes underlying: 1) ambiguity of the ripple range (100-250Hz) for dissecting IPSP-based and AP-based forms of HFOs, including their different spectral features; 2) fluctuations in the ripple and fast ripple range (>250 Hz) of individual events, resembling experimental data and 3) how AP-based HFOs could arise from asynchronous firing. This is done in the abstract and Introduction but it is somehow diluted along the text and specifically in Discusssion. Some suggestions include:

- We feel the ms would benefits from revision to avoid reiteration. For instance, section 'Pathological' HFOs are generic...' in Discussion is somehow reiterative and can be synthetized into the previous section 'Mechanisms of normal and pathological HFOs'. (see my point 23)

- We suggest moving the section 'Conclusions' to the first paragraph of Discussion to provide a succinct summary of your major novel findings, as suggested by R1.

- In addition, since some predictions are not really new authors should consider comments 2 and 3 by R2 and revise the whole ms accordingly. Regarding R2's comments on interneuronal firing rate, we discussed the picture is not crystal clear. For physiological ripples in vivo, work by Klausberger and Somogyi shows PV-basket cells can fire at stereotyped high frequency rate, but axoaxonic cells did not, neither O-LM cells. In vitro, Hajos et al found similarly for PV-basket but not for axoaxonic nor O-LM cells. Marchioni and Macaferri found some variability for epileptiforms events, as did Spampanato and Mody. So, you can take this into consideration when addressing points 2 and 3 by R2.

- We also agreed that the analytical/math section of 'Analytical proof of HFOs from asynchronous firing' in Methods is still an issue, as we commented for your previous submission. First, it breaks the narrative and advance results making the whole story difficult to follow. Second, we find this part would better fit in a more theoretical study that authors could further exploit. Finally, we would need feedback from a theoretician with expertise in neuroscience which would make the revision process longer and difficult. I would therefore ask removing this part; together with the corresponding paragraph in Results (see my points 7, 16). If the authors still feel this is an essential part of their work they should try integrating this part into the result section in a more compact manner.

- Results regarding the reconstructed LFP model could be headed independently in a new section (see my point 16). We also suggest that Fig.9, for the reconstructed LFP model, should come earlier, since it introduces the generic LFP model for both AP (left) and IPSPs (right). Then Fig.8 (AP) and Fig.9 (IPSP) could follow. This hopefully would make the narrative shorter and smoother. See my point 17

- The whole ms would benefits from carefully reading to make it a little more compact and smooth to read. See some of my comments along the pages.

2) While authors made an effort in clarifying parameters used and model definition there are some omissions:

- See point 2 by R1. In particular, we further discussed about this point in the consultation session and this is what R1 clarified about this point:

“Regarding my point 2: The parametric studies they present in Figures 8-9 has to do with how they distribute their spikes or IPSPs over time. Not the waveform itself of APs and IPSPs. This is only very briefly studied in Figure 10 where they give a few examples of t_rise and t_decay for their IPSPs. My point is that the readers should know the basic and important synaptic and AP parameters of their biophysical model (which are then also used in the constructed LFP one). We shouldnt have to reach Figure 10 to find out their default GABA decay time for example. And synaptic conductance or any AMPA-related parameter is never given in the text. These basic numbers should be presented at Methods”

- Please provide parameters used for simulations in Fig.4, as obtained from data shown in Fig.2 and 3. (See my point 11)

- Regarding justification/meaning of satellite cells, see my point 4

- Regarding LFP signals, see my points 5 and 6

- Regarding justification of parameter ranges, see my points 9, 10, 11, 13, 14

- See points 3 and 4 by R1 regarding data presentation

- Please consider whether showing representative intracellular traces for the biophysical model will help to better appreciate the role of AP, IPSPs and depolarizing block in HFOs. See my points 11 and 15

- There are some unclear sentences in the text. See minor points by R2 and my points 12, 21,

- For some clarifications and references, see my points 1,2,3, 18, 19, 20 and 22

Regarding point 1 by R2 on the need for experimental validation: we discussed and agreed this is not strictly required.

### Editor's comments:

1.Page 1, first para of Introduction: 'HFOs are brief oscillations of the local field potentials..'

2.Page 2, first para: Useful to make a note that clinical HFOs have been typically recorded with macroelectrodes (Jirsh, Urrestarazu) but also with microelectrodes (Bragin, Le Van Quyen, Worrell).

3.Page 2, second para: sparse pyramidal cell firing during ripples was described by Csicsvari and it should be cited (either the JN 1999 paper or the Neuron 2000)

4.Page 3, third para: I would like to insist on the idea that when connectivity ratios (pyr-interneurons; pyr-pyr) and the % of active cells (80/3080=2.5%), satellite and pyramidal cells can be conceptualized as the same population of pyramidal cells. Maybe a note could be helpful.

5.Page 4, biophysical model, LFP equation: It seems authors estimate laminar LFP profiles along all modeled somatodendritic compartments. However, only one LFP channel is reported at 50 um from the cell layer (according to Fig.1B). How is that LFP signal derived from the laminar LFP obtained from equation (1)? Is this reflecting a micro- or a macro-electrode? (An hippocampal pyr cell somata has about 20 um diameter, hence 3080 pyr cells would yield about 60 mm)

6.Page 4, constructed LFP model: the basic assumption for convoluting signal waveforms is linearity. This should be stated. Also useful to cite Bazelot et al J Physiol 2010 (for how micro-field IPSPs and EPSPs are generated) and Gold et al. JNeuroPhysiol 2006 for extracellular AP waveforms.

7.Page 6, section on 'Analytic proof...': I found this part breaks the rational flow, makes the ms more difficult to follow and represent a more theoretical derivation. Without this part the ms still keeps focused. I would consider leaving it out.

8.Page 8, first result section: May be useful to recall the reader the noise is uncorrelated. Also here, it may be useful to define fast ripple when calling to Fig.2A. Actually the sentence “(The peak frequency in the fast ripple band had to have higher spectral power than the peak frequency in the ripple band for at least 25% of the duration of the sharp wave in order to count as a fast ripple event.)” in page 9, second para, should probably come earlier in the first section of Results.

9.Fig.2A and 3A, why is noisy intensity range different? Can fast ripples (i.e. HFO > 250 Hz) be generated for pyr cell simulation with larger noise intensity as in Fig.2?

10.Page 9, section Effects of compromised inhibition: It would be useful to the reader to identify what is the connection with the previous set of simulations. For instance: 'We therefore investigated the effects of compromising network inhibition on fast ripple generation...starting from model shown in Fig.3 for xxx (parameters)'

11.Fig.4. Please, clarify the parameter settings used for each simulation. Maybe useful to use similar time scale in all figures. Is there any relationship between HFO spectral characteristics (or parameter regime) and duration? I also find useful to show one representative example of the intracellular signal in both pyr cell types for each simulation.

12.Page 9, same section, second para: '...until at extreme levels the network is completely uncoupled, leading to asynchronous pyramidal cell spiking. Yet, as shown in Fig. 5B, even asynchronous networks ...' Not sure where the asynchronous nature can be seen.

13.Fig.5 caption: clarify what parameter range is used (as mentioned in previous fig) to generate this simulation.

14.Page 9, section Generation of ripples...: 'To address the first question, we ran simulations in which uncorrelated noisy input was delivered to the 80 active pyramidal cells, with no other cells in the network and no coupling between pyramidal cells.' Again, it would be useful to the reader to identify what is the connection with the previous set of simulations. How does it relate to previous simulations (i.e. Fig.3 versus Fig.6)?

15.Fig.6: ? Useful to show one representative example of the intracellular signal to appreciate the nature of intracellular firing and depolarizing block.

16.Page 10, second para: This relates to the mathematical derivation that I suggest to remove. A new section for the reconstructed LFP should better follow. The word agnostic seems to be weird when referring to the model. Was not blind a better usage?

17.Fig.8,9 and 10: I suggest that Fig.9, for the reconstructed LFP model, should come earlier, since it introduces the generic LFP model for both AP (left) and IPSPs (right). Then Fig.8 (AP) and Fig.9 (IPSP) could follow. This hopefully would make the narrative shorter and smoother.

18.Page 10, last para: The evolution of the network preferred HFO frequency would depend on the intrinsic firing rate of individual cells, and this was examined in Ibarz et al.

19.Page 11 and Fig. 8: The results that the lower the jitter the larger the fast ripple occurrence seems to contradict experimental data (Foffani et al. 2007). This may be due to the nature of the generic model that considers asynchronous stationary firing, in contrast to the transient all-or-none nature of population bursts that compose HFOs. To me this just reflects the epiphenomenalism of HFOs.

20.Page 11, second para: for AP contribution to HFO the work by Csicsvari should be quoted.

21.Page 12, first para, last sentence: '...LFP are unlike to produce fast ripples..' Not sure I understand this sentence.

22.Page 13, second para: A note should be added on the major caveat with the reconstructed LFP model, i.e. that it doesn't take into account the complex spatiotemporal non-linear relationship between different somatodendritic sinks and sources that buildup LFP signals.

23.Page 14, section Pathological HFOs are generic..., could be integrated into section Mechanisms of normal and pathological..' to avoid reiteration.

24.Useful to check Schlingloff et al. JN 2014

### R1's comments:

Advances the Field (Required):

Through a series of simulations of biophysical networks or generic spiking patterns the authors explore how normal and abnormal ripple oscillations can emerge in the LFP through the network's structure or lack thereof. This yields some interesting basic insights on the underlying nature of these LFP rhythms.

Comments to the Authors (Required):

The authors have done substantial work to expand and improve the analysis of their simulations and the scope of their manuscript. I find the narrative still non-linear and somewhat convoluted (e.g. the story would perhaps be easier to follow if they would start from the constructed LFP model and then moved up in complexity with the full biophysical one instead of the other way around). This makes the line of results slightly difficult to follow conceptually. Nevertheless I believe the main results, as presented in the revised manuscript, have substantially increased the significance of the work and I would at this point support its publication.

Please take into consideration the following minor points:

1) Due to the complicated nature of the analysis and the length (and wordiness) of the manuscript itself, I think the Discussion would benefit by starting off with a brief list of main findings.

2) Since APs and IPSP waveforms are key to the both models, the authors should describe their dynamics in the Methods section (rise time, decay time, half-width duration, amplitude, synaptic conductance).

3) No error bars are ever described. Please indicate what they represent in corresponding figure legends.

4) I think Figure 5A would benefit if it was reversed and the x-axis read “Percent removed inhibitory connections” so that the curve is an increasing one. That way Panels B-D would be presented in reversed order so that the effect becomes more and more pronounced as the reader moves forward through the panels, instead of the other way around.

### R2's comments:

Advances the Field (Required):

Mitigated. Possible mechanisms able to generate a transient switch from ripple into pathological fast-ripples are particularly interesting and are an important question.

But this study is fully computational. Predictions of the model are not really new. None of the predictions were experimentally verified.

Comments to the Authors (Required):

This article is a re-submission. The authors have answered most issues raised by previous reviewers.

Using a detailed model comprising one type of interneuron (mimicking perisomatic projecting basket cell) and pyramidal cells, this study shows that action potential and IPSP can both contribute to ripples in LFPs. The model also predicts that HFOs can be produced either by coherent firing of basket cells or pyramidal neurons. Pathological ripples emerge when generated by APs and arise when synaptic input overcomes network inhibition enough to allow out of phase firing. Feed forward inhibition limits HFO in the gamma/ ripple band. Interestingly Fast Ripples could be evoked only in case of impairment of inhibitory feedback connections, inducing asynchronous firing of pyramidal neurons. FRs emerge briefly and spontaneously from asynchronous activity in an uncoupled network.

The possible mechanisms able to generate a transient switch from ripple (produced by action potentials and the loss of inhibitory network) into pathological fast ripple are particularly interesting and are an important question. The document is well referenced. Although very simplified, the hippocampus network comprises feedforward /collateral excitation and feedforward inhibition.

However some concerns remain.

1-This study is fully computational. None of the prediction was experimentally verified.

Considering that thousands of distinct configurations of detailed computational model can produce plausible biological signals (Marder E1, Taylor AL Multiple models to capture the variability in biological neurons and networks. Nat Neurosci. 2011 Feb;14(2):133-8.), produced by a very simple neuronal network, it seems important to check experimentally at least some predictions of the model. This would considerably strengthen the article.

2-From all the results cited above, most of them confirm previously published results. Several points have been already pointed out by the previous reviewers (IPSP/AP vs HFO, how inhibition shape ripple activity, role of AP in Ripple) so I just add three more:

a)“fast ripple episodes occurred due to two out-of-phase spiking clusters” as the author indicate themselves that this was already published by (Foffani et al., 2007; Ibarz et al., 2010) and also ( Demont-Guignard et al., 2012).

b)Pathological ripples emerge when enhance pyramidal cells activity is coupled with a decrease of inhibitory IPSP. This result is somehow similar to the fact that an increase of excitation of glutamatergic cells coupled to a decrease of GABAergic inhibition would generate FR as reported in (Bragin et al J Neurosci 2002 and Wendling et al, EJN2012 ).

c)Conditions leading to either ripple or fast ripple in the hippocampus, see (Aivar et al., JNeurosci, 2014)

Therefore, the authors should clearly emphasize the novel predictions and minor the already known points.

3-The authors show that in the model, HFOs were elicited through coherent firing of 20 BC cells. Peak network frequency (reaching fast ripple frequency >250Hz in the LFP) closely matches the mean basket cell firing frequency (1-300 Hz). The authors therefore explain that this LFP signal reflects IPSP due to BC firing. However, experimental data tends to show that fast spiking interneurons (as basket cells or chandelier cells of rat or monkey) barely fire above 100 Hz, when recorded in current clamp mode (Massi et al., 2012; Povysheva et al., 2013). This is an important issue. The author should provide references , contradicting the references cited above, showing that in vivo, or at least in brain slices,(in epileptic condition ?) basket cells (or other perisomatic targeting interneurons) could sustain a firing rate >150Hz.

Note that, in epileptic or non-physiologic conditions, CA3 pyramidal cells could fire at 150-300Hz as shown with simultaneous patch-clamp and field recording in hippocampal slices by (Aivar P, Valero M, Bellistri E, Menendez de la Prida L.J Neurosci 2014).

What happens when interneuronal gap junctions are suppressed in the model? Does it change the interneuronal firing rate or the emergence of ripple / FR in the LFP? It might be interesting to know if the presence interneuron gap junctions is able to change the frequency of HFO.

Minor:

P9: Results: “However, in contrast to the prior case (Fig. 2), at higher frequencies the lower frequency peak, produced by pyramidal cell action potentials, dominated.”

This sentence doesn't sound clear to me.

P9: “Thus, this model shows the transition from what are likely normal to epileptic HFOs. However, with this configuration (all connections intact) it was impossible to elicit fast ripples for two reasons [...]”

Did the author mean that “epileptic HFO” that are pathologic ripple (i.e<250Hz) ? How can the authors be sure that these oscillations are “pathologic”?
